# The making of *Tara* Oceans: funding blue skies research for our Blue Planet

**DOI:** 10.15252/msb.20156271

**Published:** 2015-05-28

**Authors:** Eric Karsenti

**Affiliations:** Scientific Director Tara Oceans

The *Tara* Oceans project emerged from an early romantic idea I had in 2000: organizing a sailing expedition in the wake of Darwin's voyage aboard the *Beagle* to popularize Biology. The original concept was mostly educational and media-oriented. Many years later, in spring 2007, with the help of a few colleagues, a scientific dimension was added to the project, which was key to start motivating funders. The long search for support and for a suitable sailing vessel was finally successful, when in fall 2007 Eric met Etienne Bourgois and agnès b., the founders of *Tara* Expeditions. This non-profit organization runs the 110-foot polar exploration schooner *Tara* since 2003, participates in scientific expeditions and promotes environmental awareness with a very efficient communication and artistic program to reach the public. Both the idea of a 3-year around-the-world expedition to study pelagic plankton organisms and the urge to raise awareness about the serious environmental issues affecting oceans appealed to the organization. The synergy that would result from combining a solid scientific concept with a vigorous communication strategy convinced both parties to engage into a long-term commitment.

Together with a few adventurous colleagues, the first scientific meeting to plan the expedition was organized in Villefranche-sur-Mer in fall 2008. Inspired by the structure and functioning of EMBL, coordinators for various specialties were appointed. Specialists for the main domains of life (viruses, bacteria, archaea, protists and metazoans) were needed, as well as oceanographers, ecologists, molecular and cellular biologists, physicists and bioinformaticians, including experts on imaging, databases and sequencing. Each of the scientists involved in this early phase recruited additional colleagues in a wonderfully self-organized process. With input from the different disciplines, we determined sampling zones, organisms' size fractions to be collected, and strategies for sample storage, handling and dispatching. Using a sophisticated bar coding system, high-quality environmental data were linked to biological samples. The early discussions were passionate and robust debates ensued. At times, we wondered whether we would make it. But with the increasing awareness of the exceptional scope of this project dedicated to the study, an entire biome at a planetary scale, a sense of great excitement and uniqueness started to diffuse throughout the growing consortium.

Despite having first-class seed funding in the form of the boat, securing further financial support for the planned scientific projects turned out to be a considerable challenge. By the nature of its approach and its goals, the project was outside of the usual boundaries of funded scientific research. Since the consortium mostly consisted of European researchers, we first solicited the European Commission, without success, as our project did not fit into existing calls, and preparation of new ones takes time. We also faced strong skepticism toward this ambitious project from both funders and reviewers. In particular, the interdisciplinary nature of the project proved to pose serious challenges to evaluators. For example, reviewers from the European Research Council felt that a cell biologist should not run an ocean project. A foundation representative with no biology background argued that the biological data collected would be of little use simply because it was not linked to some recent earth-science databases. These issues reflect some of the major challenges faced by funding bodies when evaluating interdisciplinary proposals. It remains a difficult task for funders to find reviewers with the suitable set of expertise and a mindset appreciative of high-risk ideas. While there is a broad consensus that interdisciplinarity is a driver for innovation and discovery, efficient instruments to fund such projects are sorely lacking.

In the end, it was only the collective enthusiasm and commitment of the individual participants that made *Tara* Oceans possible. Remarkably, the scientists involved in the project were so excited by the adventure that they were willing to contribute their own funding. A seemingly heteroclite number of bodies were approached: EMBL, the French Center for Atomic Energy (CEA), CNRS, the Council of Bretagne, the French Ministry of Research and all other institutions where consortium members were employed. *Tara* Expeditions furthermore negotiated private deals with various companies and organizations such as Foundation Veolia and agnès b. herself. All these manageable amounts turned out to be an efficient funding model that distributes the risk and burden among various partners, with little overhead, and is thus highly scalable. With this system, we eventually managed to secure funding for the expedition itself. Financial support for the analysis phase of the project and for the coordination of the large consortium was then partially covered by a more classical French funding program (“Investissements d'avenir” and its project OCEANOMICS, http://www.oceanomics.eu/). The 3-year expedition itself (including boat maintenance and crew) costs about 6 million Euros and the sequencing, initial imaging and bioinformatics analysis about 10 million Euros (excluding the salaries of scientists provided by institutional funding). In the initial analysis phase that started in 2011 (Sunagawa *et al*, 2015a), several meetings per year helped coordinating the various efforts and *Tara* Oceans progress was subjected to review by an international Scientific Advisory Board (http://oceans.taraexpeditions.org/en/jdb/scientific-advisory-board/) to ensure the highest scientific quality.

**Figure 1:**
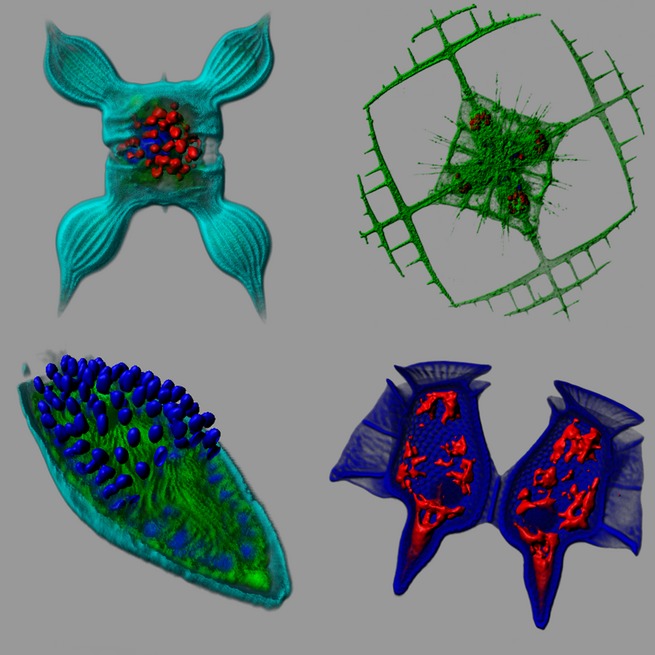
An unsuspected diversity of eukaryotic unicellular organisms (protists) was revealed by *Tara* Oceans Ribosomal DNA metabarcoding analysis and confocal laser scanning microscopy were applied to preserved plankton samples to understand the fundamentally symbiotic nature of these complex cells which often contain more genes than us humans. From upper left to down right: a diatom (*Chaetoceros* sp.) with its nucleus (blue) and chloroplasts (red); an acantharian (*Lithoptera* sp.), with skeleton structure in strontium sulfate and haptophyte endosymbiotic algae (red); a dinoflagellate infected by the parasitoid alveolate *Amoebophrya* sp.; a heterotrophic dinoflagellate (*Dinophysis* sp.) harboring kleptoplasts (red). Modified from Figure 4 in de Vargas *et al* (2015). Copyright: ©S.Colin, EPEP/SBRoscoff, CNRS & ALMF EMBL.

Fifteen years after what was initially a wild dream, a treasure trove of incredibly exciting data (Fig1) is revealed to the scientific community (Brum *et al*, 2015; Lima-Mendez *et al*, 2015; Sunagawa *et al*, 2015b; de Vargas *et al*, 2015; Villar *et al*, 2015) and, thanks to an intensive communication campaign, the *Tara* Oceans expedition has had a wide public impact worldwide (Box 1). We have demonstrated that a scientific dream off the beaten tracks can become a reality, albeit requiring a tremendous amount of motivation, collective awareness, creativity, dedication and willpower. Yet, *Tara* Oceans might serve as a model for future large-scale projects that can grow with great efficiency if they are started with a small and solid foundation that is extended, bottom-up, into a high-impact international effort by the collective contributions of the project's participants.

**Box 1 Communicating the science to the public (Coordination: Christian Sardet)**

*Tara* Oceans was not only a scientific expedition but also an exceptional human adventure. Beyond data collection and analysis, a chief objective of the expedition was to raise awareness about the fragility of marine ecosystems. Having both scientists and artists on board for long periods revived the tradition of legendary expeditions, such as Darwin's Beagle, and the Challenger. Indeed, more than 250 biologists, oceanographers, sailors, journalists, writers and artists from 40 countries took turns aboard the schooner *Tara*. Scientists and crew members met with local communities in 30 different countries during stopovers in 50 ports of call. Communicating with local people, authorities, educators and NGOs was a major task for all participants, and more than 10,000 school children were received aboard *Tara* for visits. Several large-scale media events and scientific conferences took place across the globe, including a stopover in New York organized as part of the mission to reach out to politicians at the United Nations, which culminated with the visit from the Secretary General of the United Nations, Mr. Ban Ki-moon on board *Tara*.

Further general public and educational materials are available through a multitude of channels:

- The *Tara* Oceans and *Tara* Oceans Polar Circle Web site, including its newsletters, media library and educational material.
- Social media: Facebook, Twitter.
- Science Web sites: EMBL Tara Oceans Science, Plankton Chronicles.
- YouTube shows: Tara Oceans, Tara Oceans Polar Circle Part 1, Part 2.

Books:

- “Tara Oceans, chroniques d'une expédition scientifique”, E Karsenti, D Diméo (Actes Sud, 2012).
- “Journal de bord d'une scientifique”, S Nicaud (Le Pommier, 2012).
- “Voyage autour du pôle à bord de Tara”, V Hilaire (Hachette, 2014).
- “Plancton – aux origines du vivant” (Ulmer 2013), C Sardet.
- “Plankton – wonders of a drifting world” (Univ. Chicago Press, 2015), C Sardet.
